# Gestational age‐dependent gene expression profiling of ATP‐binding cassette transporters in the healthy human placenta

**DOI:** 10.1111/jcmm.13966

**Published:** 2018-11-08

**Authors:** Guinever E. Imperio, Mohsen Javam, Phetcharawan Lye, Andrea Constantinof, Caroline E. Dunk, Fernando M. Reis, Stephen J. Lye, William Gibb, Stephen G. Matthews, Tania Maria Ortiga‐Carvalho, Enrrico Bloise

**Affiliations:** ^1^ Department of Physiology University of Toronto Toronto Ontario Canada; ^2^ Laboratory of Translational Endocrinology Institute of Biophysics Carlos Chagas Filho, Federal University of Rio de Janeiro Rio de Janeiro Brazil; ^3^ Lunenfeld‐Tanenbaum Research Institute Mount Sinai Hospital Toronto Ontario Canada; ^4^ Division of Human Reproduction, Department of Obstetrics and Gynecology Federal University of Minas Gerais Belo Horizonte Brazil; ^5^ Department of Obstetrics and Gynecology University of Toronto Toronto Ontario Canada; ^6^ Department of Medicine University of Toronto Toronto Ontario Canada; ^7^ Department of Obstetrics & Gynecology and Department of Cellular & Molecular Medicine University of Ottawa Ottawa Ontario Canada; ^8^ Department of Morphology Federal University of Minas Gerais Belo Horizonte Brazil

**Keywords:** ABC transporters, ABCA6, gestational‐age, lipid transporter, placenta

## Abstract

The ATP‐binding cassette (ABC) transporters control placental transfer of several nutrients, steroids, immunological factors, chemicals, and drugs at the maternal‐fetal interface. We and others have demonstrated a gestational age‐dependent expression pattern of two ABC transporters, P‐glycoprotein and breast cancer resistance protein throughout pregnancy. However, no reports have comprehensively elucidated the expression pattern of all 50 ABC proteins, comparing first trimester and term human placentae. We hypothesized that placental ABC transporters are expressed in a gestational‐age dependent manner in normal human pregnancy. Using the TaqMan^®^ Human ABC Transporter Array, we assessed the mRNA expression of all 50 ABC transporters in first (first trimester, n = 8) and third trimester (term, n = 12) human placentae and validated the resulting expression of selected ABC transporters using qPCR, Western blot and immunohistochemistry. A distinct gene expression profile of 30 ABC transporters was observed comparing first trimester *vs*. term placentae. Using individual qPCR in selected genes, we validated the increased expression of *ABCA1* (*P* < 0.01), *ABCA6* (*P* < 0.001), *ABCA9* (*P* < 0.001) and *ABCC3* (*P* < 0.001), as well as the decreased expression of *ABCB11* (*P* < 0.001) and *ABCG4* (*P* < 0.01) with advancing gestation. One important lipid transporter, ABCA6, was selected to correlate protein abundance and characterize tissue localization. ABCA6 exhibited increased protein expression towards term and was predominantly localized to syncytiotrophoblast cells. In conclusion, expression patterns of placental ABC transporters change as a function of gestational age. These changes are likely fundamental to a healthy pregnancy given the critical role that these transporters play in the regulation of steroidogenesis, immunological responses, and placental barrier function and integrity.

## 1 INTRODUCTION

The ATP‐binding cassette (ABC) superfamily comprise 50 proteins classified into seven sub‐families ranging from ABCA through ABCG. Although some of the superfamily members (ABCE and ABCF subfamilies) act as translation factors,^1^, ^2^ the majority of the ABC superfamily are transporters, involved in the efflux transport activity of specific substrates across biological barriers.^3^, ^4^, ^5^ In the placenta, ABC transporters regulate fetal accumulation of numerous physiological compounds, chemicals, and drugs that may be present in the maternal circulation.^6^ Endogenous substrates of the ABC transporters include steroid hormones (glucocorticoids, mineralocorticoids, estrogens, androgens, and progestogens), nutrients (lipids, cholesterol, and folate), metabolic products (oxysterols, bilirubin‐ and bile salts‐conjugated compounds) and immunological factors (cytokines and chemokines).^5^, ^7^, ^8^ Exogenous substrates include different environmental chemicals (bisphenol A, ivermectin, organochlorine, and organophosphorus pesticides) and xenobiotics (antiretrovirals, antidepressants, antibiotics, etc).^5^, ^9^, ^10^, ^11^, ^12^ Thus, placental ABC transporters control cellular metabolism, regulate local, and systemic immunological responses and orchestrate the biodistribution of numerous endogenous and exogenous substrates at the maternal‐fetal interface.^5^, ^13^, ^14^

The cellular localization of ABC transporters in the syncytiotrophoblast (eg apical *vs*. basolateral) is essential to determine the transfer orientation of their substrates in or out of the placental barrier.^5^, ^14^, ^15^, ^16^ P‐glycoprotein (P‐gp, encoded by *ABCB1*), breast cancer resistance protein (BCRP, *ABCG2*), and the multidrug resistance‐associated proteins (MRPs)‐2 and 3 (*ABCC2* and *ABCC3,* respectively) are localized to the apical membrane of syncytiotrophoblasts, indicating their extrusion activity occur from within the syncytium (and thus the fetal compartment) towards the maternal blood‐enriched intervillous space of the placenta. Conversely, ABCG1, MRP‐1, 3 and 5 (*ABCC1*,* ABCC3,* and *ABCC5*) are localized to the basolateral membrane, suggesting that extrusion activity occurs from the maternal to the fetal compartment.^5^ In some cases, the localization of ABC transporters appears to change with advancing gestation. This is the case for the ABCA1 lipid transporter, which has been associated with altered steroidogenesis, placental malformation, reduced pregnancies, and offspring morbidity.^17^, ^18^, ^19^, ^20^, ^21^

Considering that ABC transporters are also involved in the biodistribution of several drugs commonly prescribed during pregnancy (ie antiemetic agents, antibiotics, synthetic glucocorticoids, anti‐inflammatory, antidepressants, antihypertensive, and antiretroviral drugs)^5^, ^22^; a better understanding of the developmental expression of ABC transporters in the placenta may provide important insight concerning drug bioavailability into the fetal compartment throughout pregnancy. We and others have previously demonstrated a time‐dependent expression of P‐gp, BCRP, MRP‐1, 2 and 5 in the human placenta throughout pregnancy,^14^, ^23^, ^24^, ^25^, ^26^, ^27^, ^28^, ^29^, ^30^ however, there is no information about the developmental expression of other ABC transporters, highlighting the importance of a more detailed investigation of the expression pattern of the ABC transporter superfamily throughout pregnancy. We hypothesized that healthy human placentae exhibit a developmentally regulated pattern of ABC transporter mRNA expression. Therefore, we sought to comprehensively investigate the developmental expression of the ABC transporter superfamily (50 genes) comparing first trimester and term human placentae from uncomplicated pregnancies.

## 2 MATERIALS AND METHODS

### 2.1 Sample selection and study design

Healthy human first trimester (7‐9 weeks gestation, n = 8) and term (>37 weeks gestation, n = 12) placental samples (paraffin embedded slides and snap‐frozen tissues) were obtained from the Research Centre for Women's and Infants’ Health (RCWIH) BioBank, after approval of the University of Toronto's Ethics Committee (Protocol #26573). Healthy first trimester placental tissue specimens were obtained following the D&C (dilation and curettage) procedure. Placental villous tissue was visually identified and dissected from other tissues (eg decidua), by highly experienced RCWIH staff. Similarly, placental villous tissue from term pregnancies was dissected and harvested immediately after birth. Placental core sampling was performed by positioning the maternal surface facing up. Dissection was then undertaken in quadrants in areas 1.5 cm away from: the closest placental edge, the center of the placental disc, the umbilical cord insertion site, from areas of thrombosis, infarcts or other abnormalities. The cuts were made to a core depth that excluded the maternal decidua and the chorionic plate. Thus, only placental villous tissue from healthy first and term placentae were included in the study. Placental snap‐frozen tissue was stored at −80°C until the time of processing, and paraffin embedded slides were stored dry at room temperature. Considering the privacy policies regulating the collection of specimens by the biobank, first trimester patients’ clinical data were unavailable. However, term patients’ clinical data were accessible and described as follows. Maternal average age was 35 ± 1.2 years and BMI was 22 ± 1.1 kg/m^2^. Term pregnancies were 39.5 ± 0.31 weeks and an average birth weight of 3.435 (±165) g. All term samples were obtained from Caucasian mothers bearing male neonates, to minimize sample heterogeneity.

### 2.2 Total RNA extraction and cDNA synthesis

Extraction of total RNA from placental tissue (~30 mg) was performed using the Universal RNeasy Mini kit (Qiagen, Toronto, ON, Canada), in accordance with the manufacturer's instructions. Total RNA concentration was assessed using Nanophotometer and RNA integrity using Experion™ RNA Analysis kit (Bio‐Rad, Mississauga, ON, Canada). Samples were used when RNA purity (260/280 absorbance) ratio was >1.8, and RNA integrity number was >7. Total RNA (1 μg) was converted into cDNA using the SuperScript^®^ kit VILO™ cDNA Synthesis (Invitrogen, Grand Island, NY, USA).

### 2.3 ABC transporters low density array and individual qPCR

TaqMan^®^ Human ABC Transporter Array microfluidic cards (TLDA, catalog number #4378700; Applied Biosystems, Foster City, CA, USA) were used to analyse placental ABC transporters’ mRNA expression, as described previously.^31^ This array was specifically designed to assay gene expression of the 50 ABC transporters and a further 14 reference genes. Briefly, samples were run in triplicate using a total of 10 TLDA cards. Each TLDA card consisted of eight reservoirs (four per sample). A total of 400 ng of reverse transcribed mRNA was used per sample (100 ng/reservoir). cDNA was combined with the 2X Taqman^®^ Gene Expression Master Mix (final volume 100 μL/reservoir) and loaded into separate reservoirs followed by centrifugation. Cards were then sealed and run individually in an Applied Biosystems ViiA™ 7 qPCR System (technologies to Thermo Fisher Scientific, Missisauga, ON, Canada), using the following cycling conditions: 95°C for 20 seconds, followed by 40 cycles of 95°C for 1 second and 60°C for 20 seconds. The geometric mean of the three most stable reference genes (beta‐2‐microglobulin [*B2M*], Tata‐box binding protein [*TBP*] and RNA polymerase II subunit A [*POLR2A*]) was used to normalize the ABC transporter mRNA levels. Cycle thresholds were assessed performed with Thermo Fisher Cloud online software (Life Technologies), the relative expression of target genes was calculated by the 2^−ΔΔCT^ method.^32^ Heatmaps were obtained using R programming statistical software (Foundation for Statistical Computing, Vienna, Austria). A pre‐screening was undertaken, comparing samples derived from births at term by vaginal (n = 8) or cesarean section (n = 4). No differences in the relative expression of all 50 ABC transporters were observed between these two modes of delivery, thus all term placental specimens were combined into the Term group. After analysis of relative expression, the resulting *P*‐values were corrected for multiple testing by a false discovery rate (FDR)^33^ of 5% using the R programming statistical software.

To validate the relative expression results obtained in the array, individual qPCR of selected ABC genes, *ABCA1*,* ABCA6*,* ABCA9*,* ABCB1, ABCB11*,* ABCG4,* and *ABCC3,* was assessed using the same Taqman^®^ probes present in the TLDA cards (ID: Hs00194045_m1, Hs00365329_m1, Hs00184824_m1, Hs00184491_m1, Hs00358656_m1, Hs00223446_m1 and Hs00329320_m1, respectively). Their relative expression was normalized using the gene *POLR2A* (Hs00172187_m1). qPCR reactions were using the Taqman^®^ Universal Master Mix II (Applied Biosystems) in triplicates in a CFX96 real‐time PCR detection system (Bio‐Rad). The cycling conditions were: 50°C for 2 minutes, 95°C for 10 minutes, followed by 40 cycles of 95°C for 15 seconds and 60°C for 60 seconds. Changes in mRNA expression were calculated according to the 2^−ΔΔCT^ method.^32^

### 2.4 Western blot

To investigate protein expression of the selected ABC transporter, total protein was extracted from placental tissue (~50 mg) using approaches described previously.^31^ Briefly, nitrocellulose membranes to which protein had been transferred were incubated overnight at 4°C in the presence of a specific primary antibody for the proteins of interest: Anti‐ABCA6 (ab180567; Abcam, Toronto, ON, Canada) in a 1:250 PBS dilution with 5% BSA; and anti‐ERK (sc‐7383; Santa Cruz Biotechnology, Dallas, TX, USA) as an internal control, diluted 1:3000 in PBS with 5% milk. The membrane was then washed, processed, and analysed as described previously.^31^

### 2.5 Immunohistochemistry

Mounted paraffin‐embedded tissue sections (0.5 μm thickness) were processed as described previously.^31^ Briefly, antigen retrieval was performed by incubating pre‐heated (3 minutes in microwave) sections with target retrieval solution, pH 9 (Dako Agilent Technologies, Mississauga, ON, Canada) (2 × 20 minutes on ice); followed by an incubation in sodium citrate solution (10 mmol/L 2 × 15 minutes on ice). Slides were then incubated with Protein Block (Dako) for 1 hour, followed by an overnight 4°C incubation with the primary antibodies: ABCA6 (Abcam, 1:250) and anti‐IgG (Dako, used to replace the primary antibody, as a negative control). Sections were then washed in PBS (3 × 5 minutes) and incubated with anti‐mouse IgG secondary antibody (X0931; Dako) for 1 hour at room temperature, followed by incubation with streptavidin‐HRP (1 hour; Dako) and visualized using diaminobenzidene (Dako). Slides were counterstained with haematoxylin, dehydrated and cover slipped. Sections were examined using an Olympus BX61 upright, motorized microscope coupled with an Olympus DP72 digital camera (Olympus, Tokyo, Japan) at 20X magnification.

### 2.6 Statistical analysis

Prism software (GraphPad Software Inc., San Diego, CA, USA) version 5.0 was used for statistical analysis. Analysis included the Kolmogorov‐Smirnov normality test followed by an unpaired Student's‐*t* test or the non‐parametric Mann‐Whitney test. A 5% FDR was applied to the array data to correct for multiple comparisons. The data displayed refers to the adjusted *P*‐value after FDR correction. Specific statistical analysis and the number of samples used are described in the legend of respective figures. Results are expressed as mean ± SEM. Differences were considered significant when *P* was <0.05.

## 3 RESULTS

### 3.1 Time‐dependent gene expression of ABC transporters in the human placenta

**Figure 1:**
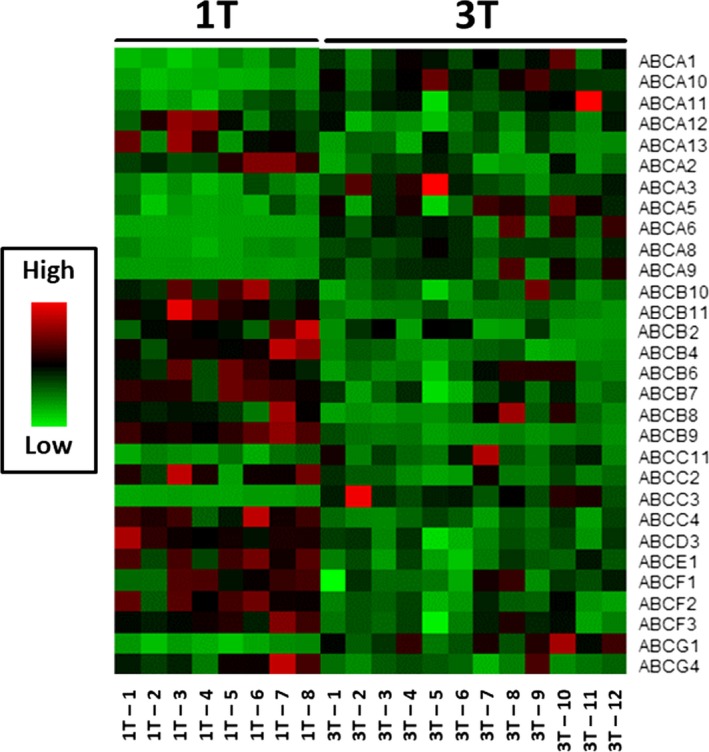
Heatmap indicating the time‐dependent expression of the 30 ATP‐binding cassette (ABC) genes that exhibited developmentally regulated expression in healthy human placentae. Relative quantities (Rq) of the ABC genes obtained from the Human ABC Transporters Taqman^®^ Array, comparing first trimester (n = 8) and term (n = 12) human placentae. Red represents increased expression, green indicates reduced expression

**Figure 2:**
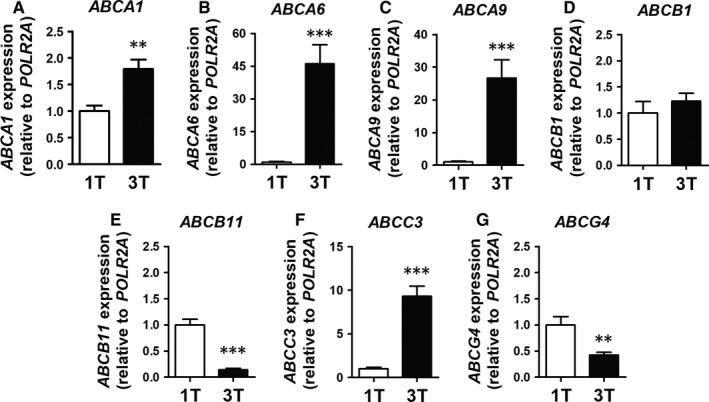
Validation of selected ABC genes by qPCR. (A) *ABCA1*; (B) *ABCA6*; (C) *ABCA9*; (D) *ABCB1* (E); *ABCB11*; (F) *ABCC3*; and (G) *ABCG4* mRNA expression, comparing first trimester (n = 8) and term (n = 12) placentae. Statistical analyses: non‐parametric Mann‐Whitney test. Data are presented as mean ± SEM. **P < 0.01 and ***P < 0.001

We observed a dramatic difference in gene expression between first trimester and term placentae, clearly visible in the heatmap (Figure 1) and detailed in Table 1. Healthy placental development was associated with downregulation of 18, and upregulation of 11 ABC transporters (Table 1). *ABCA13*,* ABCB4*,* ABCB9*,* ABCB11*,* ABCC2,* and *ABCG4* were the most decreased, while *ABCA6*,* ABCA8*,* ABCA9*,* ABCA10*,* ABCC3,* and *ABCG1* are the most increased ABC genes (Table 1). As previously observed,^31^ expression of *ABCG5* and *ABCG8* was below detection limit and, therefore, not included in the analysis. *ABCB5*,* ABCC8*,* ABCC12,* and *ABCC13* transcripts exhibited inconsistent amplification results likely because of very low levels of expression, and were not evaluated. Based on their potential physiological relevance and mRNA abundance (baseline mRNA expression), seven ABC transporter genes were selected for validation using individual qPCR. We confirmed the same pattern previously observed in the array, i.e., significantly increased expression of *ABCA1* (*P* < 0.01), *ABCA6* (*P* < 0.001), *ABCA9* (*P* < 0.001) and *ABCC3* (*P* < 0.001), no difference in *ABCB1* and significantly decreased expression of *ABCB11* (*P* < 0.001) and *ABCG4* (*P* < 0.01), in first trimester compared to term placentae (Figure 2).

**Table 1:** Fold‐change in mRNA expression of the ATP‐binding cassette (ABC) transporters, obtained from the Human ABC Transporters Taqman^®^ Array, comparing term (n = 12) to first trimester (n = 8) human placentae

Name	Fold‐change	Name	Fold‐change	Name	Fold‐change	Name	Fold‐change
*ABCA1*	2.14***	*ABCA12*	0.56*	*ABCB11*	0.16***	*ABCD1*	1.18
*ABCA2*	0.62**	*ABCA13*	0.42*	*ABCC1*	1.06	*ABCD2*	1.60
*ABCA3*	2.38**	*ABCB1*	0.73	*ABCC2*	0.48***	*ABCD3*	0.62***
*ABCA4*	0.68	*ABCB2*	0.59**	*ABCC3*	4.13***	*ABCD4*	0.92
*ABCA5*	1.73**	*ABCB3*	0.92	*ABCC4*	0.55***	*ABCE1*	0.64***
*ABCA6*	2.63***	*ABCB4*	0.28***	*ABCC5*	1.12	*ABCF1*	0.76**
*ABCA7*	1.25	*ABCB6*	0.69**	*ABCC6*	1.29	*ABCF2*	0.64***
*ABCA8*	3.74**	*ABCB7*	0.65***	*ABCC7*	1.42	*ABCF3*	0.74***
*ABCA9*	3.02***	*ABCB8*	0.83*	*ABCC9*	0.77	*ABCG1*	2.73***
*ABCA10*	4.45***	*ABCB9*	0.33***	*ABCC10*	1.07	*ABCG2*	0.80
*ABCA11*	1.64*	*ABCB10*	0.58**	*ABCC11*	1.83*	*ABCG4*	0.47**

Fold‐change was calculated as the ratio between term and first trimester expression. Fold‐change >1 indicates increased expression; <1 indicates decreased expression.

*P < 0.05, **P < 0.01 and ***P < 0.001.

### 3.2 ABCA6 placental protein expression increases at term and is localized to the syncytiotrophoblast

**Figure 3:**
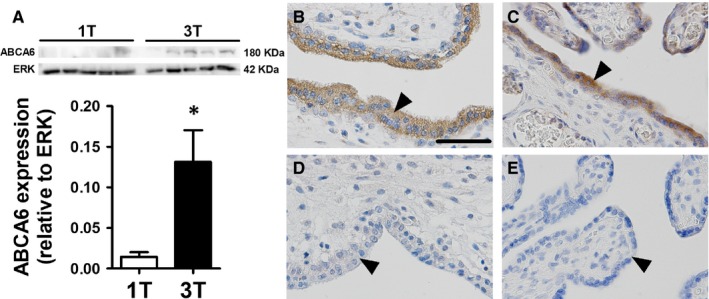
Human placental ABCA6 protein expression and immunolocalization towards term. (A) Representative ABCA6 (180 kDa) and ERK (42 kDa) immunoblots, comparing first trimester (n = 5; open bar) and term (n = 5; solid bar) human placentae, as well as placental ABCA6 expression normalized to ERK (loading control). (B and C) Representative images of ABCA6 localization in first trimester and term placentae, respectively. (D and E) Negative controls for the respective gestational age. Statistical analyses: unpaired *t*‐test. Data are presented as mean ± SEM *P < 0.05, arrows indicate the syncytium, scale bar: 55 μm

We next evaluated whether protein levels of a specific ABC transporter, would follow the same pattern as for gene expression. We selected an as yet uncharacterized ABC transporter, the ABCA6 lipid tranporter for analysis, based on its degree of change (fold change: 2.63, *P* < 0.001) and potential physiological relevance for the placenta. As shown in Figure 3A, ABCA6 exhibited increased (*P* < 0.05) total protein expression at term, demonstrating correspondence between increased transcription and translation of this tranporter with advancing gestation. Immunohistochemical analysis indicated that ABCA6 protein was highly localized to the cytoplasm of the syncytiotrophoblast. Variable ABCA6 immunoreactivity in the microvillous membrane of the syncytiotrophoblast was also detected, though at a much lower level compared to the cytoplasmic staining. Additionally, some cells of interstitial villi of both first trimester and term placentae were also positive for ABCA6 (Figure 3B,C).

## 4 DISCUSSION

In the current study, we show for the first time, a gestational‐age specific pattern of expression of 30 ABC transporters in the healthy human placenta, highlighting a potential role for these transporters in regulating placental development, metabolism, and intrauterine disposition of specific substrates throughout pregnancy. Importantly, the lipid transporter ABCA6 exhibited increased placental mRNA and protein expression towards term and was highly localized to the syncytiotrophoblast in first trimester and term placentae, suggesting that ABCA6 may have a relevant role supporting placental lipid metabolism and transport, particularly in late pregnancy, when protein levels are at their highest.

Among all the regulated ABC genes, the ABCA subset exhibited the greatest degree of upregulation across pregnancy, particularly for *ABCA6*,* ABCA8*,* ABCA9,* and *ABCA10*. This is perhaps unsurprising, considering that this subfamily of transporters comprises the major lipid transporters responsible for lipid homeostasis,^34^, ^35^, ^36^ lipid translocation and cell signalling.^37^, ^38^, ^39^, ^40^ In fact, ABCA transporters are of primary importance for cholesterol transport, which is paramount for normal fetal brain and overall body development.^41^, ^42^, ^43^, ^44^, ^45^, ^46^, ^47^, ^48^ Cholesterol is also the precursor of lipid metabolites such as oxysterols and steroid hormones.^49^, ^50^, ^51^, ^52^ Given that the human placenta lacks the cellular machinery required for cholesterol synthesis,^53^ and the fact that cholesterol can originate from both fetal and maternal compartments,^54^, ^55^ the presence of developmentally‐regulated cholesterol transporters in trophoblast cells is likely essential for placental biology. Interestingly, we detected a 2.14‐fold increased expression in *ABCA1* mRNA at term compared to first trimester. This finding contrasts with previous published data demonstrating no changes in placental *ABCA1* mRNA levels comparing early *vs*. late pregnancy placentae.^20^ These differences may be due to patient selection criteria and or differing sensitivity of the techniques undertaken in both studies.

We selected the ABCA6 lipid transporter for further protein analysis. *ABCA6* exhibited a marked gestational time‐dependent regulation and to the best of our knowledge, has never been previously characterized in the placenta. Placental ABCA6 protein levels increased with advancing gestation, which paralleled increases in mRNA levels detected by the microfluid array and individual qPCR. Furthermore, ABCA6 was highly localized to the syncytiotrophoblast cytoplasm regardless of the gestational age. Some sections exhibited variable localization in the microvillous membrane of the syncytiotrophoblast and in interstitial villi cells in both first trimester and term placentae. Considering that ABCA6 is expressed at Golgi complexes,^40^ our results suggest that ABCA6 may contribute to lipid homeostasis in the syncytiotrophoblast, probably by transferring cholesterol and oxysterols across the Golgi complex membrane. Furthermore, the variable presence of ABCA6 in the microvillous membrane of the syncytiotrophoblast suggests that it may also contribute to lipid transport in the placental barrier across pregnancy. However, these hypotheses require further investigation.

Levels of mRNA expression of ABCG genes involved in lipid transport and homeostasis were also developmentally regulated. Of importance, *ABCG1* was highly upregulated in term placentae. ABCG1 has been demonstrated to synergize with ABCA1—which also exhibited higher gene transcript levels in term placentae, to efflux cholesterol and generate HDL particles.^56^

In contrast to the ABCA subset of transporters, the ABCB transporters exhibited the greatest decreases of mRNA throughout pregnancy. Nine out 11 ABCB transporters were decreased at term, in particular, *ABCB2*,* ABCB4,* and *ABCB9* mRNA. The ABCB subset comprise transporters known to elicit multidrug resistance to neoplastic cells.^57^ In the placenta, ABCB transporters confer embryo/fetal protection against xenobiotics and environmental toxins that may present in the maternal circulation.^5^, ^14^ The best well‐characterized placental ABCB transporters are *ABCB1,* which encodes P‐gp and *ABCB4,* the MRP‐3 encoding gene.^5^ Previously, we have demonstrated a distinct pattern of time‐dependent expression of P‐gp/*ABCB1* in the human placenta. P‐gp is highly expressed in the human placenta and is primarily localized to the apical membrane of the syncytiotrophoblast. P‐gp functions as a major efflux transporter that protects the fetus from accumulation of several obstetric‐relevant drugs and environmental toxins^5^ and is an important transporter that modulates extravillous trophoblast invasion in early pregnancy.^58^ In our previous studies, syncytiotrophoblast P‐gp staining and placental *ABCB1* expression decreased towards term, suggesting a higher protection of the fetus from exposure to P‐gp substrates in the first trimester, a period in which the developing conceptus is most vulnerable to teratogenicity.^23^, ^59^ Our present findings did not recapitulate the previous observed reduced placental *ABCB1* expression towards term. This result from several factors, including patient selection criteria, different specificity of qPCR techniques undertaken, different reference genes used (*POLR2a*,* TBP* and *B2M vs*. *YWHAZ*,* HPRT* and *SDHA*), the evaluation of distinct *ABCB1* transcripts: according to Ensembl Genome Database Project (http://www.ensembl.org/), the human *ABCB1* gene has 11 transcripts (splice variants), with four of them being protein coding, which may have led to the evaluation of different *ABCB1* transcripts in these studies. Nevertheless, reduced expression of nine ABCB transporters’ genes supports the notion that placental‐mediated fetal protection to certain substrates declines in late gestation. In parallel with these decreases in the placenta, there is a dramatic increase in P‐gp in the developing fetal blood‐brain barrier which confers protection of the brain during the fetal to neonatal transition.^5^, ^60^, ^61^

*ABCB4 (MDR3)*, along with the other downregulated ABC transporters including the bile salt export pump *ABCB11* (BSEP), *ABCC2 (*MRP2) and *ABCC4 (*MRP4), are components of the hepatobiliary‐like excretory system.^62^, ^63^, ^64^ These transporters provide an important route of elimination of toxic compounds produced by fetal metabolism, such as bile acids,^63^, ^65^ that otherwise due to the immaturity of the fetal liver would accumulate and harm the fetus.^66^ Similarly, placental expression of the peroxisomal transporter *ABCD3,* which is involved in fatty acids oxidation and bile acids synthesis^67^ was decreased at term compared to first trimester. Our current results support a previous study,^62^ that suggested placental excretory activity is gradually replaced by fetal hepatic performance with the development and maturation of the fetal liver. This highlights the potential importance of ABC transporters for the clearance control of bile acids and conjugates of bilirubin, bile salts and xenobiotics,^5^, ^68^, ^69^, ^70^, ^71^, ^72^, ^73^ particularly in earlier stages of pregnancy.

Gene expression of the intracellular ABC proteins, ABCE1 and ABCF1‐3, was also decreased in term placentae. While their placental function are not known, our data provide evidence these ABC genes may developmentally regulate intracellular processes in trophoblast cells, which may include ribosomal cycling and protein synthesis^74^ and inflammatory responses to cytosolic DNA (DNA sensing)^75^ and chorioamnionitis.^31^

*ABCG2*, which encodes the multidrug resistance transporter BCRP, did not show changes in gene expression throughout pregnancy. Like P‐gp, BCRP is highly expressed in the apical membrane of the syncytiotrophoblast barrier to confer fetal protection against harmful substances present in the maternal blood; but with an additional role, to regulate cytotrophoblast fusion into syncytiotrophoblasts.^76^ Stable levels of *ABCG2* across pregnancy are consistent with our previous studies.^23^, ^27^ BCRP staining however, is increased in the syncytiotrophoblast towards the end of pregnancy, indicating an increment of fetal protection against BCRP substrates as gestation proceeds.^23^, ^27^

In conclusion, placental development is associated with a very specific expression pattern of 30 ABC transporter genes. We have also demonstrated a gestational age‐dependent pattern of ABCA6 mRNA and protein expression and its abundant localization to the syncytiotrophoblast. Our findings suggest that ABCA6 is likely to exert yet unexplored gestational‐age dependent actions in placental lipid homeostasis and transport. Our data also highlight the need for further studies exploring the role of other yet uncharacterized developmentally‐regulated ABC transporters in the placenta, which likely exert important actions throughout pregnancy. Considering their crucial role in regulating steroidogenesis, placental nutrient transfer, barrier efficiency and integrity, as well as diverse intracellular processes, the ABC transporters in the placenta likely play critical roles in normal, and pathological pregnancies. This represents a critical area for future research.

## AUTHOR'S ROLES

Conception and design: GEI, WG, TMOC, SGM, EB. Acquisition of data, analysis and interpretation of data: GEI, MJ, PL, AC, CD, EB. Drafting the article or revising it critically for important intellectual content: GEI, FMR, WG, SJL, TMOC, SGM, EB. Final approval of the version to be published: GEI, MJ, PL, AC, CD, FMR, WG, SJL, TMOC, SGM, EB.

## Conflict of Interest

None declared.
